# Automated Caries Screening Using Ensemble Deep Learning on Panoramic Radiographs

**DOI:** 10.3390/e24101358

**Published:** 2022-09-24

**Authors:** Toan Huy Bui, Kazuhiko Hamamoto, May Phu Paing

**Affiliations:** 1 Course of Science and Technology, Graduate School of Science and Technology, Tokai University, Tokyo 108-8619, Japan; 2 Graduate School of Information and Telecommunication Engineering, Tokai University, Tokyo 108-8619, Japan; 3 School of Engineering, King Mongkut’s Institute of Technology Ladkrabang, Bangkok 10520, Thailand

**Keywords:** caries screening, dental radiographs, ensemble, deep learning

## Abstract

Caries prevention is essential for oral hygiene. A fully automated procedure that reduces human labor and human error is needed. This paper presents a fully automated method that segments tooth regions of interest from a panoramic radiograph to diagnose caries. A patient’s panoramic oral radiograph, which can be taken at any dental facility, is first segmented into several segments of individual teeth. Then, informative features are extracted from the teeth using a pre-trained deep learning network such as VGG, Resnet, or Xception. Each extracted feature is learned by a classification model such as random forest, k-nearest neighbor, or support vector machine. The prediction of each classifier model is considered as an individual opinion that contributes to the final diagnosis, which is decided by a majority voting method. The proposed method achieved an accuracy of 93.58%, a sensitivity of 93.91%, and a specificity of 93.33%, making it promising for widespread implementation. The proposed method, which outperforms existing methods in terms of reliability, and can facilitate dental diagnosis and reduce the need for tedious procedures.

## 1. Introduction

Dental health is important because of the correlation between oral health problems and illnesses such as cardiovascular disease and diabetes. Oral health has a significant impact on their overall health and quality of life. Oral health problems such as mouth and face discomfort, oral and throat cancer, oral infection and sores, periodontal (gum) diseases, tooth decay, and tooth loss impede a person’s ability to bite, chew, and speak and affect psychological health. In 2016, the World Health Organization (WHO) projected that over 3.5 billion individuals were impacted by oral disorders and expected this number to continue to rise [1].

**Figure 1:**
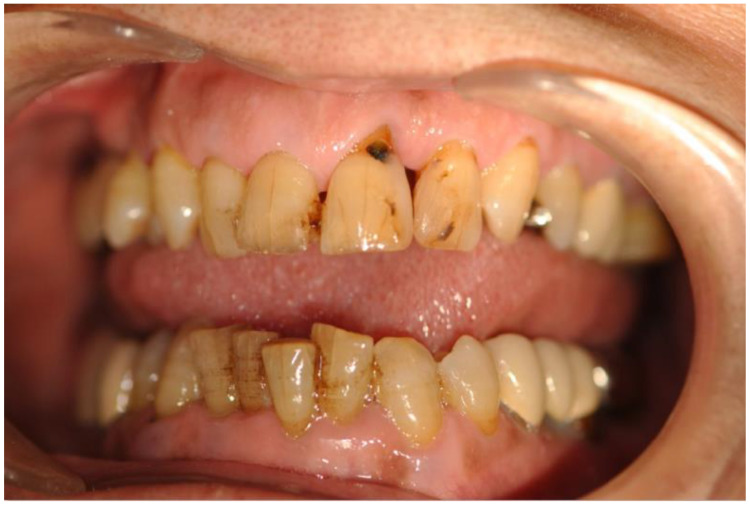
Caries in teeth.

Dental caries form when acids produced by bacteria in the mouth erode dentin, causing damage to tooth structure or attachment, which can make gums bleed. They are the most common chronic oral disease in adults, affecting around 60% of adults over the age of 50. Dental health is part of oral health [2], including the state of oral tissues as well as factors that can affect oral health. Dental plaque is initially a soft, thin film. Soft plaque turns into hard plaque, which cannot be easily removed by brushing, via mineralization with calcium, phosphate, and other minerals [3]. Over time, caries cause holes, destroy the tooth, and increase the risk of further damage, including tooth loss (Figure 1).

Medical imaging technology, such as that based on X-rays and other forms of radiation, is used for diagnosis and treatment. Multimodal medical imaging technologies allow more than one form of radiation to be used at the same time to obtain an image that is more accurate and complete. Such technologies help doctors determine the best course of action for their patients. They also help reduce pain and speed up the diagnosis process. A concern of patients is radiation exposure. However, the radiation emitted is generally very low-level and is not likely to cause any long-term health problems.

Advancements in medical imaging technology enable the rapid gathering and analysis of a large amount of data. Computer-aid diagnoses (CADs) can assist physicians to interpret 2D and 3D images [4]. 3D imaging provides more detail and is thus useful for complex cases. A deep-learning-based method can segment the mandible from core beam computed tomography images [5]. 2D imaging provides essential information for diagnosing problems such as cancer, diabetes, and caries [6,7]. Several studies [8,9,10] have advocated the use of photoacoustic images, wavelength images, or ultrasound imaging for caries detection. Other studies [10,11] have proposed an approach that employs an RGB oral endoscope image. However, most systems cannot observe the detailed anatomy of a tooth, especially the root, and hence cannot be used to diagnose caries. Dental radiography is a simple and affordable imaging method that can be performed in most dental offices and hospitals; other imaging techniques, such as CT radiography and near-infrared ranging, are more costly and thus less commonly used [12]. Dental radiography images are thus preferable for the early detection of caries based on computer-aid diagnosis.

## 2. Literature Review

Caries detection based on radiography uses panoramic radiographs, periapical images, bitewing images, or occlusal images. Panoramic radiographs, which are the most complex, present the health condition of all teeth and provide a benefit of medical history in a whole oral image, whereas the other types of images show only a few teeth in a specific region. Periapical, bitewing, and occlusal images provide similar information. Therefore, panoramic radiographs are more informative and preferred for caries detection 

Li et al. [13] used support vector machine (SVM) and a backpropagation neural network (BPNN) to identify tooth decay. The autocorrelation coefficient and the gray level co-occurrence matrix are used separately in their method for feature extraction. SVM and BPNN models are then used separately for classification. On a testing set, SVM had an accuracy of 79% and BPNN had an accuracy of 75%. These accuracies are insufficient for practical applications. Their study did not describe the dataset and thus the validity of their research is unknown.

Yu et al. [14] attempted to improve the backpropagation neural network layer and autocorrelation coefficient matrix feature extraction. Their approach was evaluated using 80 private dental radiographs. An accuracy of 94% was obtained; however, as the number of network layers increases, the system becomes more computationally expensive. The sensitivity, specificity, precision, and F-measure were not reported. The small testing data (35 photographs) and lack of cross-validation are shortcomings of their study.

Patil et al. [15] developed a dragonfly-specific intelligent system. The feature set is extracted using multi-linear principal component analysis (MPCA). After the characteristics are loaded into a neural network classifier, the classifier is trained using the adaptive dragonfly algorithm as an optimization strategy. 120 private dental photographs were used to assess the MPCA model non-linear programming with the adaptive dragonfly algorithm (MNP-ADA) with three test scenarios. Each test case consisted of a total of 40 photographs, 28 and 12 of which were utilized for training and testing, respectively. Other classifiers and feature sets, such as linear discriminant analysis (LDA) [16], principal component analysis (PCA) [17], and independent component analysis (ICA) [18], as well as fruit fly (FF) [19] and grey-wolf optimization (GWO) [20], were employed for comparison. The MNP-ADA model achieved an accuracy of 90%, a sensitivity of 94.67%, and a specificity of 63.33%. This low specificity indicates that patients without caries were incorrectly labeled as patients with caries. The high precision but limited specificity may raise questions about the data balance between photographs with and without caries.

Singh et al. [21] proposed an automated caries detection method based on Radon transform (RT) and discrete cosine transform (DCT). To capture low-frequency information, RT is performed on X-ray images for each degree. 2D DCT is then applied to the RT images to extract frequency characteristics (DCT coefficients). These characteristics are transformed into a 1D coefficient vector in a zigzag way. Principal component analysis is then applied to this vector to retrieve features. Using decision tree, k-nearest neighbor, random Forest, naive Bayes, sequential minimum optimization, radial basis function, decision stumps, and AdaBoost classifiers, the smallest number of features are then combined. The best result was achieved with random forest, with an accuracy of 86%, a sensitivity of 91%, and a specificity of 80%.

Le et al. [22] proposed a framework for diagnosing dental problems, called the Dental Diagnosis System (DDS), that uses panoramic radiographs. It is based on a hybrid approach that combines segmentation, classification, and decision-making. For the segmentation task, it used the best method for dental image segmentation, which based on semi-supervised fuzzy clustering. For the classification task, a graph-based algorithm called affinity propagation clustering was developed. To select a disease from a group of diseases found in the segments, a decision-making method was developed. DDS was designed based on actual dental cases in Hanoi Medical University, Vietnam, which included 87 dental photographs of cases with five prevalent diseases, namely root fracture, wisdom teeth, tooth decay, missing teeth, and periodontal bone resorption. The accuracy of DDS is 92.74%, which is higher than those of systems based on fuzzy inference (89.67%), fuzzy k-nearest neighbor (80.0%), prim spanning tree (58.46%), Kruskal spanning tree (58.46%), and affinity propagation clustering (90.01%). Their dataset consisted of various types of images, which may have led to unreliable results.

Most previous researches have an undependable method which is low performance or cannot fully automated diagnosis. In the present study, we comprehensively evaluate panoramic radiographs and develop a fully automated and dependable caries screening approach.

## 3. Material and Method

### 3.1. Dataset

**Figure 2:**
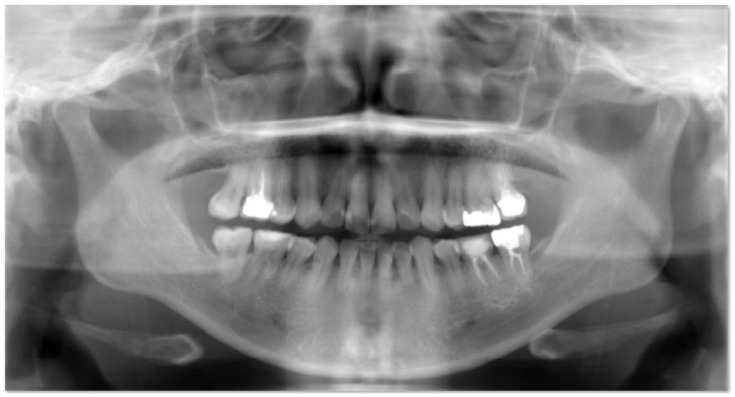
Panoramic radiographs.

We received a dataset from dentists at Shinjuku East Dental Office. The dataset consists of unprocessed radiographs of 95 individuals. These radiographs were automatically processed to generate 533 tooth regions in the tooth region proposal stage. Images are from real patient cases from the hospital. The patients were 18 years old or older and provided consent. It is important to highlight that caries is more severe in adults (over 18 years old) since their teeth are no longer milk teeth but rather permanent teeth, which cannot be restored to their previous state. The University Committee at Tokai evaluated the publishing and usage rights of the images in the dataset based on ethical considerations. Figure 2 shows an example image from the dataset. It includes the mouth and a portion of the patient’s jaw bone.

### 3.2. Method

**Figure 3:**
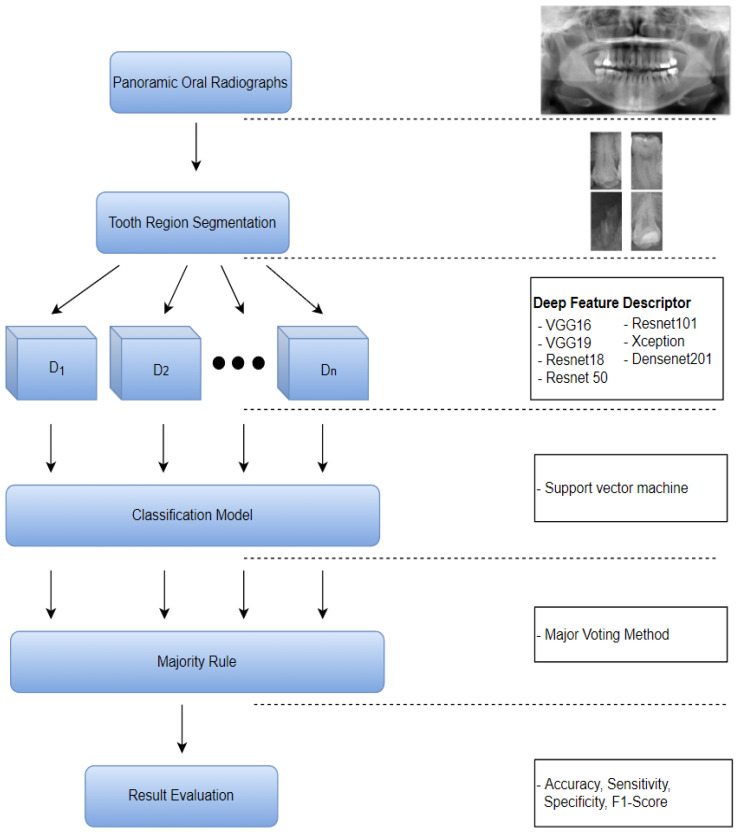
Diagram of the proposed method.

The proposed method, shown in Figure 3, consists of tooth segmentation, tooth feature descriptor, and caries prediction processes. In the first stage, a YOLO model is applied for tooth region proposal. Then, the proposal region is segmented from the image and fed into the feature descriptor. Several pre-trained networks, namely VGG16 [23], VGG19 [23], Resnet18 [24], Resnet50 [24], Resnet101 [24], Xception [25], and Densenet201 [26], are used as feature descriptors to extract informative features. Next, the features are used to train an SVM [27] classifier. Finally, a majority voting method is applied using the model features to produce the final optimal result.

#### 3.2.1. Tooth Region Segmentation

As mentioned, caries detection methods that directly use images received from the dentist have been developed. The images are usually either unprocessed or periapical images, which makes using them expensive in terms of human labor and cost. In the present research, an automatic region proposal method is used to reduce cost and improve diagnosis.

**Figure 4:**
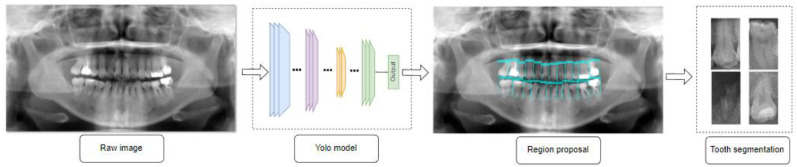
Tooth segmentation process.

First, we create an image’s region of interest. To prevent encroachment on the teeth, we choose a region in the center of the image with a preliminary ratio compared to the original image of 1:1.4. The images are scaled to fit the Yolov3 model’s input size. The YOLOv3 model is used to suggest a tooth region, with Squeeze Net as the network’s base [28,29,30]. We increase the number of detection heads and concatenate the output of each detection head with a suitable layer to generate better results. However, we must consider the model’s size to avoid overfitting and decrease complexity. Three detection heads are utilized in this detection model. A detailed illustration of the tooth segmentation process is shown in Figure 4. The fine-tuned parameters are given in Table 1.

#### 3.2.2. Deep Pre-Trained Network as Feature Descriptors

In this work, a convolutional neural network with pre-trained weights is employed as a feature descriptor to extract deep activated features. To determine the best descriptor of pre-trained networks, the seven most popular networks, namely VGG16, VGG19, Resnet18, Resnet50, Resnet101, Xception, and Densenet, were used. Technically, the network processes RGB pictures, whereas the radiographs are grayscale; hence, we multiplied the grayscale channel to replace the image’s missing channels. Table 2 shows the depth, parameters, size, and input size for each pre-trained model. Among the network models, Densenet has the most layers (201), and VGG16 has the fewest layers (23).

#### 3.2.3. Classification

The extracted feature set from each feature descriptor in the preceding stage is used to train an SVM classifier to predict caries [31]. The SVM model seeks to identify the ideal hyperplane for describing the difference between data (caries and non-caries in this scenario. The Gaussian radial basis function is used in the classifier to reduce the number of training points. For data $D=\left\{\left(x_{i},y_{i}\;\right),i=1\ldots N\right\}$ and $y_{i}\in \;\left\{-1,1\right\}$, the SVM model and mapping function of the Gaussian kernel can be described as follows:

(1) $$\underset{\omega ,b,\;\xi }{\min}\frac{1}{2}{\left|\left|W\right|\right|}^{2}+C\underset{i}{\sum }\xi_{i}^{2}\;subject\;to\;y_{i}\left(W^{T}X_{i}+b\right)\geq 1-\xi_{i},\;\xi_{i}\geq 0,\;\forall i$$

where *C* > 0 is the selected parameter and *ξ* is a set of slack variables.

(2) $$K\;\left(X,\;Y\right)=e^{\frac{{\left|\left|X-Y\right|\right|}^{2}}{A}}$$

where K is the kernel function and *A* is a constant.

We also applied the feature set to k-nearest neighbor [32,33] and random forest [34,35,36,37] classifiers for comparison with support vector machine.

#### 3.2.4. Majority Voting

The predictions of each feature and the SVM predictor are considered as individual opinions that depend on different contributions of accuracy performance. To produce a final prediction, voting is conducted among the predictors. The final diagnosis is made based on majority voting and compared to each individual prediction. The computation of the final prediction based on the actual prediction probability of each individual opinion is conducted as follows:

(3) $$P\left(y=j|x\right)=\frac{\exp\left[\sum_{n=1}^{N}{\hat{P}}_{n}\left(y=j|x\right)\right]}{\exp[\sum_{l=1}^{L}\sum_{n=1}^{N}{\hat{P}}_{n}(y=j|x)\;}$$

where *N* is the number of predictors, *n* is the predictor number, *L* is the number of classes, and *P* is probability.

## 4. Measures and Result Assessment

### 4.1. Measures

The performance of the proposed method was evaluated in terms of accuracy (*ACC*), sensitivity (*SEN*), and specificity (*SPEC*). In addition, the positive predictive value (*PPV*), negative predictive value (*NPV*), F1-score, and processing time are presented. The detailed calculation of each measure is as follows:

(4) $$ACC=\frac{TP+TN}{TP+FP+TN+FN}$$

(5) $$SEN=\frac{TP}{TP+FN}$$

(6) $$SPEC=\frac{TN}{TN+FP}$$

(7) $$PPV=\frac{TP}{TP+FP}$$

(8) $$NPV=\frac{TN}{TN+FN}$$

(9) $$F1-score=\frac{2TP}{2TP+FP+FN}$$

where true positive (*TP*) indicates the number of caries images correctly classified as caries, true negative (*TN*) indicates the number of non-caries images correctly classified as non-caries, false positive (*FT*) indicates the number of non-caries images incorrectly classified as caries, and false negative (*FN*) indicates the number of caries images incorrectly classified as non-caries.

### 4.2. Result Evaluation

An analysis of majority voting for several pre-trained neural networks and a classifier was conducted. The results are shown in Table 3. Overall, SVM has the best performance for every feature descriptor and in the final vote. An increase in the depth of a network increased accuracy. For SVM, the accuracy, sensitivity, and specificity with Densenet were 90.57%, 95.65%, and 86.67%, respectively, which is predictable due to the depth of the network. VGG16 had the lowest accuracy, sensitivity, and specificity (79.25%, 73.91%, and 83.33%, respectively). The majority voting made use of each feature descriptor and increased performance to 92.45% for accuracy, 95.65% for sensitivity, and 90% for specificity using an SVM classifier. Even though there might be some circumstances which random forest have a better sensitive, other measuring elements are not compatible.

**Figure 5:**
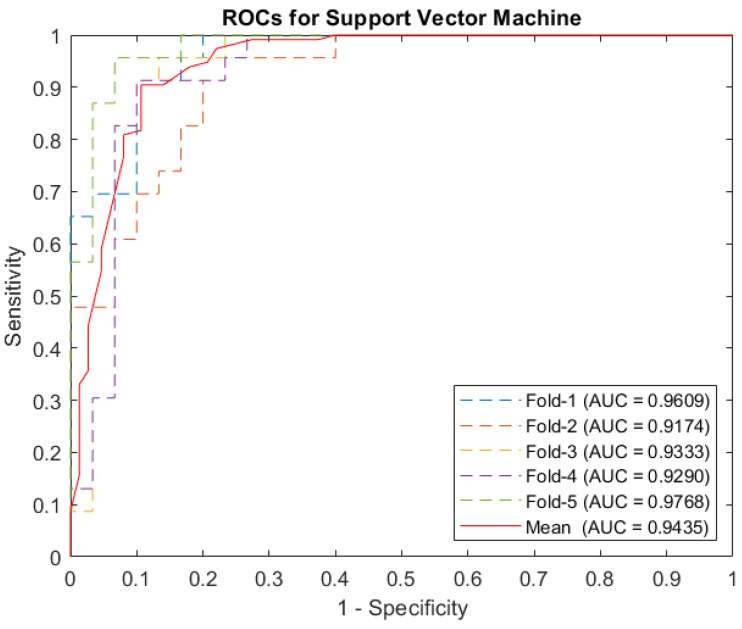
ROCs curve for support vector machine.

To develop and evaluate an effective caries detection system, the training and testing sets were randomly divided for cross-validation. The k-fold cross-validation was used to evaluate the proposed method’s robustness. The results demonstrate that the proposed method reliably adapts to unknown samples and covers the whole problem space. Additionally, k-fold cross-validation was used to avoid overfitting the proposed method to our testing data. It was applied to the method that best represents the issue, which is the SVM. The difference in accuracy between folds is around 6% (lowest accuracy: 90.57%, highest accuracy: 96.23%). All average values of accuracy, sensitivity, and specificity are higher than 93%, which indicates that our method is stable and reliable. We also computed the receiver operating characteristic (ROC) curves and area under the curves (AUC). The ROC curves, which describe the data for each fold and the average value, are presented in Table 4 and Figure 5.

To compare the complexity of the method for various feature descriptors, we computed the execution time of each process in MATLAB2020a running in a Windows 10 environment on a computer with an Intel i7 CPU and a GeForce GTX 2060 GPU, 32 GB RAM. Table 5 shows the execution time for each function in seconds. The operation for the Densenet feature descriptor is the most time-consuming. It took 113.7 seconds to finish, which is at least 10 times longer than any other operation. In comparison, the fastest process on Resnet18 took only 4.33 seconds. Without considering the training process, the proposed method can be widely used because of its high processing speed.

Finally, we compare the proposed method with state-of-the-art methods. A short description of each existing method and its dataset is given below. Existing methods use distinct datasets, whose size and complexity affect performance. Therefore, this comparison is preliminary. The specifications of the state-of-the-art methods are given in Table 6. Although the method in [15] achieved a promising 90% accuracy, its low specificity of 63.33% is insufficient. In addition, the methods in [14,15] primarily use periapical images, which are often basic and require human effort to produce the final result. In contrast, the method in [21] has a general outcome that is not particular to the state of carious teeth. Despite a promising accuracy of 92.47%, the method in [22] is hampered by its use of mixed data, which leads to unknown validity. In addition, the sensitivity and specificity of this method were not reported. The table indicates that the proposed method has an accuracy of 95.38% and outperforms most existing methods. In addition, we present a full technique evaluation in a comprehensive dataset.

## 5. Conclusions

This study proposed a method for segmentation and caries diagnosis for caries screening. Most existing methods perform caries classification using periapical images, which require human labor to extract the input image. In contrast, the proposed method extracts the tooth region of interest automatically. Although the automatically segmented images may contain some errors, the proposed method has an accuracy of 93.58%, outperforming state-of-the-art methods.

Because features are extracted from seven feature descriptors, redundant features may be overcrowded. In future work, we would like to analyze each feature’s contribution to lowering the computational cost.

## Figures and Tables

**Table 1:** Parameter of Yolo model.

Parameter	Value
Mini batch size	32
Number of anchor box	11
Iteration	1000
Initial learning rate	0.001
L2regularization	0.0005

**Table 2:** Description of pre-trained model.

Network Name	Depth	Size (MB)	Parameter (×10^6^)	Input Size
VGG16	23	528	138.4	227 × 227 × 3
VGG19	26	549	143.7	224 × 224 × 3
Resnet18	18	45	11.5	224 × 224 × 3
Resnet50	50	98	25.6	224 × 224 × 3
Resnet101	101	171	44.7	224 × 224 × 3
Xception	126	88	22.9	224 × 224 × 3
Densenet201	201	77	88.9	299 × 299 × 3

**Table 3:** Performance assessments of different models.

Classifier	Measure	VGG16	VGG19	Resnet18	Resnet50	Resnet101	Xception	Densenet	Voting
Random Forest	Accuracy	0.4717	0.4528	0.5472	0.4528	0.3774	0.4906	0.4528	0.4528
	Sensitivity	0.9565	1.0000	0.8696	1.0000	0.8261	1.0000	1.0000	1.0000
	Specificity	0.1000	0.0333	0.3000	0.0333	0.0333	0.1000	0.0333	0.0333
	PPV	0.4490	0.4423	0.4878	0.4423	0.3958	0.4600	0.4423	0.4423
	NPV	0.7500	1.0000	0.7500	1.0000	0.2000	1.0000	1.0000	1.0000
	F1-score	0.4400	0.4423	0.4545	0.4423	0.3654	0.4600	0.4423	0.4423
K-nearest Neighbor	Accuracy	**0.7925**	0.6981	0.7547	0.7736	0.8679	0.7736	0.6415	0.8491
	Sensitivity	0.6957	0.6087	0.6957	0.6087	0.6957	0.6087	0.4783	0.6957
	Specificity	0.8667	0.7667	0.8000	0.9000	1.0000	0.9000	0.7667	0.9667
	PPV	0.8000	0.6667	0.7273	0.8235	1.0000	0.8235	0.6111	0.9412
	NPV	0.7879	0.7188	0.7742	0.7500	0.8108	0.7500	0.6571	0.8056
	F1-score	0.5926	0.4667	0.5517	0.5385	0.6957	0.5385	0.3667	0.6667
Support Vector Machine	Accuracy	**0.7925**	**0.8868**	**0.8302**	**0.8679**	**0.8868**	**0.8491**	**0.9057**	**0.9245**
	Sensitivity	0.7391	0.9130	0.8261	0.8696	0.8261	0.7391	0.9565	0.9565
	Specificity	0.8333	0.8667	0.8333	0.8667	0.9333	0.9333	0.8667	0.9000
	PPV	0.7727	0.8400	0.7917	0.8333	0.9048	0.8947	0.8462	0.8800
	NPV	0.8065	0.9286	0.8621	0.8966	0.8750	0.8235	0.9630	0.9643
	F1-score	0.6071	0.7778	0.6786	0.7407	0.7600	0.6800	0.8148	0.8462

Highest values are presented in bold.

**Table 4:** Support vector machine on k-fold cross validation.

Measure	Fold-1	Fold-2	Fold-3	Fold-4	Fold-5	Mean
Accuracy	0.9623	0.9245	0.9245	0.9057	0.9623	0.9358
Sensitivity	0.9565	0.9565	0.9565	0.9130	0.9130	0.9391
Specificity	0.9667	0.9000	0.9000	0.9000	1.0000	0.9333
PPV	0.9565	0.8800	0.8800	0.8750	1.0000	0.9183
NPV	0.9667	0.9643	0.9643	0.9310	0.9375	0.9528
F1-score	0.9565	0.9166	0.9166	0.8936	0.9545	0.9276
AUC	0.9609	0.9174	0.9333	0.9290	0.9768	0.9346

**Table 5:** Execution time for each function.

Function Name	Time(s)
Load data	0.78
VGG16 + SVM training	10.35
VGG19 + SVM training	10.89
Resnet18 + SVM training	4.33
Resnet50 + SVM training	6.20
Resnet101 + SVM training	8.21
Xception + SVM training	10.53
Densenet + SVM training	113.7
Voting and Prediction	0.40
Total	165.39

**Table 6:** Comparison to previous state-of-the-art.

References	Method	Samples	ACC%	SEN%	SPEC%
[14,15]	Auto-correlation coefficients matrix Neural network	120 periapical images	73.33	77.67	53.33
[15]	Multi-linear principal component analysis Non-linear programming with adaptive dragonfly algorithm Neural network	120 periapical images	90.00	94.67	63.33
[21]	Radon transformation Discrete Cosine transformation Principal component analysis Random forest	93 panoramic images	86.00	91.00	80.00
[22]	Semi-supervised fuzzy clustering Graph-based clustering	87 mixed panoramic and periapical images	92.47	-	-
Proposed method	Deep activated features Geometric features SVM classification	95 panoramic images (533 tooth region images)	93.58	93.91	93.33

## Data Availability

Restriction applies to the availability of these data. The data were obtained from Shinjuku East Dental Office (the director is Makoto Kumon) and are available from authors with permission of Makoto Kumon or by sending a request to Makoto Kumon at: http://www.shinjukueast.com/doctor-staff/ (accessed on 3 August 2022).

## References

[1] World Health Organization Oral Health. https://www.who.int/health-topics/oral-health/.

[2] Gift H.C., Redford M. (1992). Oral Health and The Quality Of Life. Clin. Geriatr. Med..

[3] Hennessy B.J. (2021). Caries. https://www.msdmanuals.com/professional/dental-disorders/common-dental-disorders/caries.

[4] Kim S.H., Kim K.B., Choo H. (2022). New Frontier in Advanced Dentistry: CBCT, Intraoral Scanner, Sensors, and Artificial Intelligence in Dentistry. Sensors.

[5] Lo Giudice A., Ronsivalle V., Spampinato C., Leonardi R. (2021). Fully automatic segmentation of the mandible based on convolutional neural networks (CNNs). Orthod. Craniofacial Res..

[6] Mosquera-Lopez C., Agaian S., Velez-Hoyos A., Thompson I. (2015). Computer-Aided Prostate Cancer Diagnosis From Digitized Histopathology: A Review on Texture-Based Systems. IEEE Rev. Biomed. Eng..

[7] Mansour R.F. (2017). Evolutionary Computing Enriched Computer-Aided Diagnosis System for Diabetic Retinopathy: A Survey. IEEE Rev. Biomed. Eng..

[8] Sampathkumar A., Hughes D.A., Kirk K.J., Otten W., Longbottom C. All-optical photoacoustic imaging and detection of early-stage dental caries. Proceedings of the 2014 IEEE International Ultrasonics Symposium.

[9] Hughes D.A., Girkin J.M., Poland S., Longbottom C., Cochran S. Focused ultrasound for early detection of tooth decay. Proceedings of the 2009 IEEE International Ultrasonics Symposium.

[10] Usenik P., Bürmen M., Fidler A., Pernuš F., Likar B. (2014). Near-infrared hyperspectral imaging of water evaporation dynamics for early detection of incipient caries. J. Dent..

[11] Maslak E., Khudanov B., Krivtsova D., Tsoy T. Application of Information Technologies and Quantitative Light-Induced Fluorescence for the Assessment of Early Caries Treatment Outcomes. Proceedings of the 2019 12th International Conference on Developments in eSystems Engineering (DeSE).

[12] Angelino K., Edlund D.A., Shah P. (2017). Near-Infrared Imaging for Detecting Caries and Structural Deformities in Teeth. IEEE J. Transl. Eng. Health Med..

[13] Li W., Kuang W., Li Y., Li Y., Ye W. Clinical X-ray Image Based Tooth Decay Diagnosis using SVM. Proceedings of the 2007 International Conference on Machine Learning and Cybernetics.

[14] Yu Y., Li Y., Li Y.-J., Wang J.-M., Lin D.-H., Ye W.-P. Tooth Decay Diagnosis using Back Propagation Neural Network. Proceedings of the 2006 International Conference on Machine Learning and Cybernetics.

[15] Patil S., Kulkarni V., Bhise A. (2019). Intelligent system with dragonfly optimisation for caries detection. IET Image Process..

[16] Loog M., Duin R.P.W. (2004). Linear dimensionality reduction via a heteroscedastic extension of LDA: The Chernoff criterion. IEEE Trans. Pattern Anal. Mach. Intell..

[17] Lazcano R., Madroñal D., Salvador R., Desnos K., Pelcat M., Guerra R., Fabelo H., Ortega S., López S., Callicó G.M. (2017). Porting a PCA-based hyperspectral image dimensionality reduction algorithm for brain cancer detection on a manycore architecture. J. Syst. Archit..

[18] Montefusco-Siegmund R., Maldonado P.E., Devia C. Effects of ocular artifact removal through ICA decomposition on EEG phase. Proceedings of the 2013 6th International IEEE/EMBS Conference on Neural Engineering (NER).

[19] Pan W.-T. (2012). A new Fruit Fly Optimization Algorithm: Taking the financial distress model as an example. Knowl.-Based Syst..

[20] Mirjalili S., Mirjalili S.M., Lewis A. (2014). Grey Wolf Optimizer. Adv. Eng. Softw..

[21] Singh P., Sehgal P. Automated caries detection based on Radon transformation and DCT. Proceedings of the 2017 8th International Conference on Computing, Communication and Networking Technologies (ICCCNT).

[22] Tuan T.M., Fujita H., Dey N., Ashour A.S., Ngoc V.T.N., Chu D.T. (2018). Dental diagnosis from X-ray images: An expert system based on fuzzy computing. Biomed. Signal Process. Control.

[23] Simonyan K., Zisserman A. (2014). Very Deep Convolutional Networks for Large-Scale Image Recognition. arXiv.

[24] Wu S., Zhong S., Liu Y. (2018). Deep residual learning for image steganalysis. Multimed. Tools Appl..

[25] Chollet F. Xception: Deep Learning with Depthwise Separable Convolutions. Proceedings of the 2017 IEEE Conference on Computer Vision and Pattern Recognition (CVPR).

[26] Huang G., Liu Z., Van Der Maaten L., Weinberger K.Q. Densely connected convolutional networks. Proceedings of the IEEE Conference on Computer Vision and Pattern Recognition.

[27] Cortes C., Vapnik V. (1995). Support-vector networks. Mach. Learn..

[28] Redmon J., Divvala S., Girshick R., Farhadi A. You Only Look Once: Unified, Real-Time Object Detection. Proceedings of the IEEE Conference on Computer Vision and Pattern Recognition.

[29] Iandola F., Han S., Moskewicz M., Ashraf K., Dally W., Keutzer K. (2016). SqueezeNet: AlexNet-level accuracy with 50x fewer parameters and <0.5 MB model size. arXiv.

[30] Bui T.H., Hamamoto K., Paing M.P. (2022). Tooth Localization using YOLOv3 for Dental Diagnosis on Panoramic Radiographs. IEEJ Trans. Electron. Inf. Syst..

[31] Vapnik V. (1995). The Nature of Statistical Learning Theory.

[32] Fix E., Hodges J.L. (1989). Discriminatory Analysis. Nonparametric Discrimination: Consistency Properties. Int. Stat. Rev./Rev. Int. Stat..

[33] Altman N.S. (1992). An Introduction to Kernel and Nearest-Neighbor Nonparametric Regression. Am. Stat..

[34] Tin Kam H. Random decision forests. Proceedings of the 3rd International Conference on Document Analysis and Recognition.

[35] Irle A., Kauschke J. (2011). On Kleinberg’s Stochastic Discrimination Procedure. Pattern Anal. Mach. Intell. IEEE Trans..

[36] Kleinberg E.M. (1996). An overtraining-resistant stochastic modeling method for pattern recognition. Ann. Stat..

[37] Kleinberg E.M. (2000). On the algorithmic implementation of stochastic discrimination. IEEE Trans. Pattern Anal. Mach. Intell..

